# A combined bioinformatics and functional metagenomics approach to discovering lipolytic biocatalysts

**DOI:** 10.3389/fmicb.2015.01110

**Published:** 2015-10-13

**Authors:** Thorsten Masuch, Anna Kusnezowa, Sebastian Nilewski, José T. Bautista, Robert Kourist, Lars I. Leichert

**Affiliations:** ^1^Department of Microbial Biochemistry, Institute of Biochemistry and Pathobiochemistry, Ruhr University BochumBochum, Germany; ^2^Junior Research Group for Microbial Biotechnology – Department for Biology and Biotechnology, Ruhr University BochumBochum, Germany

**Keywords:** metagenomics, functional metagenomics, biocatalyst, esterase, lipase

## Abstract

The majority of protein sequence data published today is of metagenomic origin. However, our ability to assign functions to these sequences is often hampered by our general inability to cultivate the larger part of microbial species and the sheer amount of sequence data generated in these projects. Here we present a combination of bioinformatics, synthetic biology, and *Escherichia coli* genetics to discover biocatalysts in metagenomic datasets. We created a subset of the Global Ocean Sampling dataset, the largest metagenomic project published to date, by removing all proteins that matched Hidden Markov Models of known protein families from PFAM and TIGRFAM with high confidence (*E*-value > 10^-5^). This essentially left us with proteins with low or no homology to known protein families, still encompassing ~1.7 million different sequences. In this subset, we then identified protein families *de novo* with a Markov clustering algorithm. For each protein family, we defined a single representative based on its phylogenetic relationship to all other members in that family. This reduced the dataset to ~17,000 representatives of protein families with more than 10 members. Based on conserved regions typical for lipases and esterases, we selected a representative gene from a family of 27 members for synthesis. This protein, when expressed in *E. coli*, showed lipolytic activity toward para-nitrophenyl (pNP) esters. The *K*_m_-value of the enzyme was 66.68 μM for pNP-butyrate and 68.08 μM for pNP-palmitate with *k*_cat_/*K*_m_ values at 3.4 × 10^6^ and 6.6 × 10^5^ M^-1^s^-1^, respectively. Hydrolysis of model substrates showed enantiopreference for the R-form. Reactions yielded 43 and 61% enantiomeric excess of products with ibuprofen methyl ester and 2-phenylpropanoic acid ethyl ester, respectively. The enzyme retains 50% of its maximum activity at temperatures as low as 10°C, its activity is enhanced in artificial seawater and buffers with higher salt concentrations with an optimum osmolarity of 3,890 mosmol/l.

## Introduction

The global market of industrial enzymes is estimated to have a value between $ 4.8 billion and $ 5.1 billion and rising (BCC Research, 2014; The Freedonia Group, 2014). The biggest market share falls to hydrolytic enzymes like proteases, amylases, esterases, and lipases (Gupta et al., 2004). Lipolytic enzymes like lipases and esterases are of special interest for the industry, because of their various applications in organic chemistry (Jaeger and Eggert, 2002). They are able to economically outcompete some conventional chemical methods, based on their high enantioselectivity, substrate specificity, and mild reaction conditions (Divakar and Manohar, 2007).

Microbial lipolytic enzymes, esterases (EC 3.1.1.1) and lipases (EC 3.1.1.3), are ubiquitous enzymes that hydrolyze organic ester bonds in aqueous solutions. The former hydrolyze small molecules with ester bonds, which are at least partly soluble in water, whereas the latter display a maximum of activity toward long-chained and insoluble triglycerides (Jaeger et al., 1999).

The active site of lipolytic enzymes is a catalytic triad comprised of a nucleophilic serin residue, an acidic residue and a histidin residue (Brady et al., 1990; Lowe, 1992). The structure around the nucleophilic serin residue is called the catalytic elbow with a G-x-S-x-G motif, where × stands for an arbitrary residue. This catalytic elbow is the most conserved structure in this protein family (Mala and Takeuchi, 2008). In some cases the first glycin in this motif can be substituted by alanine, as for example in Lipase A from *Bacillus subtilis* (Dartois et al., 1992). The so-called oxyanion hole, which occurs in one of three variants: HGx, HGGGx, or Y is another highly conserved feature, by which lipolytic enzymes can be classified (Pleiss et al., 2000; Widmann et al., 2010).

As a result of the steady growth of the biocatalyst market there is a desire for enzymes that can either catalyze the production of new organic compounds through novel reaction pathways or work more efficient in established processes. To accomplish these goals and obtain better biocatalysts known enzymes can be modified via random or targeted mutation. Thus it was possible to modify characteristics like enantioselectivity (Qian and Lutz, 2005), activity and thermal stability (Suen et al., 2004) as well as substrate specificity (Reetz et al., 2005) of lipolytic enzymes.

These procedures need a basis in form of an enzyme which already has promising attributes. Therefore the search for novel enzymes which exhibit such attributes is a worthwhile endeavor. One especially promising prospect for the search for new biocatalysts are metagenomes with their vast amount of genes from non-cultivable bacteria. It is predicted that as few as 1% of all bacteria can be cultivated under laboratory conditions (Amann et al., 1995), which leaves a huge amount of possibly interesting proteins out of reach of classical genomics (Tringe and Rubin, 2005). DNA from environmental samples is either being sequenced and the sequencing results saved in databases for further investigations via bioinformatics as was done with the results from the Global Ocean Sampling (GOS; Rusch et al., 2007; Yooseph et al., 2007), GOS, or it is transformed into an organism for expression to construct an environmental DNA library which can be screened for the desired activity. The latter has been used frequently to search for novel lipolytic enzymes (e.g., Henne et al., 2000; Lee et al., 2006; Nacke et al., 2011; Berlemont et al., 2013; Mokoena et al., 2013). This method has also been used to discover enzymes from often difficult-to-cultivate extremophiles (Rhee et al., 2005; Tirawongsaroj et al., 2008; Levisson et al., 2009). Lipolytic enzymes are probably the most frequent targets of metagenomic expression library screens (Simon and Daniel, 2011). A wide variety of diverse habitats has been examined and lipolytic enzymes could be found in forest soil, marine sediment and water, landfill leachate, and activated sludges, to name just a few examples. Nevertheless, in light of the ever-growing (meta-)genomic sequence information available, one could argue that we have only scratched the surface of the extent of enzyme biodiversity. For a comprehensive list of reported metagenomically derived esterases and lipases see Martínez-Martínez et al. (2013).

A downside of the aforementioned method is the sheer expense of resources that has to be invested into the construction and screening of these environmental gene libraries. Typical studies performed to find novel enzymes can encompass up to 3 million distinct clones (Nacke et al., 2011). Here we present an alternative approach that has the potential to reduce the needed manpower and resources for finding novel enzymes using metagenomic *in silico* data. This approach uses a bioinformatical arrangement of metagenomic proteins into clusters of high homology, identification of one representative for each cluster and functional annotation based on known structural characteristics of the biocatalyst in question. Using the GOS dataset as a source, we were able to identify a novel cluster of lipases with comparatively low homology to known lipases. The representative of this cluster shows a broad substrate range, high activity at moderate to low temperatures and halophilic properties.

## Materials and Methods

### Annotation of Predicted Proteins with Known Domains

To identify predicted proteins with known domains, all GOS sequences were analyzed for domains from PfamA, PfamB, and TIGRFAM using the hmmsearch utility from HMMER 3.0 with an inclusion threshold *E*-value of 10^-3^ and a reporting threshold of 10^-5^. Domain Hidden Markov Models (HMMs) were obtained from ftp.ebi.ac.uk/pub/databases/Pfam/ and ftp://ftp.jcvi.org/pub/data/TIGRFAMs/ (Pfam 27, TIGRFAM 14; Haft et al., 2003; Finn et al., 2014).

### Identification of Protein Families *de novo*

The remaining 1.7 million sequences lacking known domains identifiable via HMMs were grouped using cd-hit hierarchical clustering in three steps (90, 75, 60% similarity), sequences which could not be matched with other sequences in this way were discarded. To minimize redundancy, only sequences which resulted from cd-hit clustering by 90% similarity were used. Similarity distances between these sequences were determined by all-vs.-all protein BLAST. On the basis of all similarity distances the sequences were clustered with MCL at *i* = 1.4 according to van Dongen and Abreu-Goodger (2011).

### Determination of Cluster Representatives

For each *de novo* family, guide trees generated by MAFFT FFT-NS-2 Strategy were used to estimate the similarity distance between individual family members (Katoh et al., 2002). The sequence with the smallest total distance to all other sequences was chosen as the representative for a family.

### Search for Lipolytic Enzymes

A regular expression pattern search for the active site of lipases and esterases was performed on all representatives from families lacking HMM annotation which consisted of more than 10 sequences. The regular expression sequences for the three lipase superfamilies were derived from the annotated multiple alignment of the Lipases Engineering Database (Pleiss et al., 2000; Widmann et al., 2010) and are stated in **Table 1**.

**Table 1:** Regular expression patterns for the identification of carboxylester hydrolases used in this study.

Superfamily	Regular expression pattern
GX	“/G[EFGHILMNRSTVWY].{10,}[AG].(S[AFHKLMQTVWY]| C[FLWY])G.{10,}[DE].{10,}H/”
GGGX	“/GG([AELNRSW]| G[FL]).{10,}[AG].S[ACFGIMSVY]G.{10,}[DE].{10,}H/”
Y	“/Y.{10,}[AG].S[ANQWY]G.{10,}[DE].{10,}H/”

The regular expression patterns define a necessary requirement for lypolytic activity but on its own are not sufficient. These sequences can, and do, randomly occur in unrelated proteins. We thus applied the set of HMMs of all families listed in the Lipase Engineering Database to the matching sequences to select for proteins that contain the pattern in the context of a typical hydrolase fold. The protein gi135157357 was the only candidate that matched these patterns with an *E*-value ≤ 10^-5^. This potential lipase from the GOS dataset (PL-GOS) was selected and the corresponding gene synthesized (GeneArt, Regensburg, Germany). The 3D-structure of PL-GOS was predicted using Phyre2 (Kelley et al., 2015). To determine, if the MCL clustering-based family of PL-GOS is indeed phylogenetically distinct from known lipases, a phylogenic NJ-tree tree (Kuraku et al., 2013) was created based on a MAFFT alignment of the PL-GOS family members with members of protein family abH 15.03 (sequences obtained from the Lipase Engineering Database) and abH 15.02 as outgroup. MAFFT strategy G-INS-I was used. The tree was visualized using Dendroscope 3 in a radial phylogram. abH 15.02 is not shown and was used to define the root.

### Protein Expression and Purification

The *PL-GOS* gene was cloned into a pTAC-MAT-Tag-2 (Sigma–Aldrich, St. Louis, MO, USA) based vector with an ompA signaling sequence for periplasmic expression and a pET-11a (Merck Millipore, Billerica, MA, USA) derivative for overexpression and purification via his-tag (see **Table 2**). The vectors were transformed into *Escherichia coli* strains MG1655 (periplasmic expression) and BL21 (DE3) (overexpression), respectively, by means of heat-shock transformation creating the strains listed in **Table 2**. All *E. coli* strains were grown in LB-medium (Bertani, 1951; without glucose), supplemented with 100 mg L^-1^ ampicillin at 37°C. Expression of protein in strain TMMGPK and TMMGP1503 was induced by addition of 1 mM IPTG to the medium 2 h prior to cell disruption for qualitative *para*-nitrophenyl ester (pNP) assays with crude cell extract and in TMBL1503 4 h prior to cell disruption for protein purification at an OD_600_ of 0.5.

**Table 2:** Strains of *Escherichia coli* used in this study.

*E. coli* strain	Genotype	Source
MG1655	F^-^ λ^-^ *ilvG*^-^ *rfb*-50 *rph*-1	ATCC strain 700926, American Type Culture Collection
BL21 (DE3)	F^-^ *ompT*^-^ *gal*^-^ *dcm*^-^ *lon*^-^ *hsdSB* (r_B_^-^ m_B_^-^)λ (DE3 (*lacI lacUV*5-T7 gene 1 *ind1 sam7 nin5*))	Stratagene, Santa Clara, CA, USA
TMMGP	MG1655 pPC (pTAC-MAT-Tag-2 with N-terminal *OmpAss*)	This work
TMMGPK	MG1655 pTM1 (*Bacillus subtilis lipA* in pPC)	This work
TMMGP1503	MG1655 pTM2 (*pl-gos* in pPC)	This work
TMBL1503	BL21 (DE3) pTM3 (*pl-gos* with N-terminal His6-Tag and TEV cleavage site in pEt-11a)	This work

Cells for crude extract for pNP-ester assays were harvested for 30 s at 13,000× *g*. The pellet was resuspended in 50 mM tris-HCl-buffer, pH 7.3. Crude extract was obtained by cell disruption using a Vial Tweeter (Hielscher, Teltow, Germany) at 80% amplitude in 0.5 s intervals for 3 × 1 min and subsequent spinning down of the insoluble cell debris for 20 min at 13,000× *g* and 4°C.

For the purification of PL-GOS cells were harvested by centrifugation for 30 min at 4°C and 7,800× *g*. The pellet was resuspended in 100 ml 50 mM tris-HCl-buffer, pH 7.3 per 10 g wet weight including Complete EDTA-free Protease Inhibitor Cocktail (Roche, Mannheim, Germany) (one tablet per 80 ml solution). Next, cells were disrupted three times using a Constant Cell Disruption System (Constant Systems, Low March, UK) at 1.9 kbar. The lysate was again supplemented with one tablet of Complete EDTA-free Protease Inhibitor Cocktail per 80 ml solution and 1 mM PMSF. The cell lysate was subsequently separated from cell debris for 1 h at 4°C and 7,800× *g* by centrifugation. Supernatant was filtered using 250 ml Filtropur V50 vacuum filters (Sarstedt, Nümbrecht, Germany). Purification and fractionation steps were performed with an ÄKTApurifier (GE Healthcare, Uppsala, Sweden). Filtrate was loaded onto a 5 ml HisTrap HP Ni-NTA-column (GE Healthcare, Uppsala, Sweden) equilibrated with buffer A (50 mM sodium phosphate, 300 mM NaCl, pH 8.0). Elution was performed using 150 ml of a gradient from 98% buffer A and 2% buffer B (=buffer A containing 500 mM imidazole) to 100% buffer B at a flow rate of 1.67 ml min^-1^. 3 ml fractions were collected and analyzed by SDS-PAGE. Fractions with the highest protein yield were unified and his_6_-tagged TEV protease was added to the target protein at a 30-fold excess. The protein solution was dialyzed against 50 mM HEPES-buffer in a Spektra/Por Dialysis Membrane 12–14 kDa (Spectrumlabs, Rancho Dominguez, USA) at 4°C. Next the protein solution was again loaded onto a Ni-NTA-column and eluted with buffer A to remove the his_6_-tagged TEV protease and the residual his_6_ tag. The flow-through was once again dialyzed against 50 mM HEPES-buffer and concentrated using 10 kDa filter concentrators (Santorius Stedim, Göttingen, Germany). A purity of >95% was achieved as assessed by SDS-PAGE.

### Lipolytic Activity Assays

Hydrolytic activity toward triglycerides was tested using LB agar plates containing 100 mg L^-1^ ampicillin, 100 μM IPTG and either 1% (w/v) tributyrin or 1% (w/v) triolein and 0.001% (w/v) Rhodamin B (Sigma–Aldrich, St. Louis, MO, USA). MG1655 colonies were incubated for 2 days at 37°C. Hydrolytic activity was indicated by clear halos around the colonies for tributyrin and under UV light orange fluorescent halos for triolein.

Lipolytic activity was tested by using pNP-esters (pNP-butyrate, C4; pNP-octanoate, C8; pNP-decanoate, C10; pNP-duodecanoate, C12; pNP-myristate, C14, and pNP-palmitate, C16) (Sigma–Aldrich, St. Louis, MO, USA) as substrates. A reaction sample containing 65 nM purified PL-GOS or 1% (v/v) crude extract in 50 mM tris-HCl-buffer, pH 7.3 was kept at a constant temperature. The reaction was started by adding 200 μM pNP-ester from a 4 mM stock in aqueous solution with 4% ethanol, 1% acetonitrile, and 0.4% Triton-X100 and was observed by measuring the OD_405_ of the sample over the course of 10 min. To analyze the temperature optimum pNP-butyrate was used as a substrate and reactions were repeated at different temperatures ranging from 10 to 45°C. The salt concentration optimum was detected by replacing tris-HCl-buffer, pH 7.3 with artificial seawater (28.13 g/l NaCl; 3.5 g/l MgSO_4_⋅7 H_2_O; 2.55 g/l MgCl_2_; 1.2 g/l CaCl_2_; 0.77 g/l KCl; 0.11 g/l NaHCO_3_) (Atlas, 2010) using pNP-butyrate as the substrate at a temperature of 20°C. Different osmolarities were established by adding or withholding NaCl. For measurement of the Michaelis Menten kinetics, the reaction was performed in tris-HCl-buffer, pH 7.3 with different substrate concentrations of pNP-butyrate and pNP-palmitate ranging from 5to 333 μM.

For activity assays with crude extract the TMMGPK strain expressing lipase A from *B. subtilis* was used as positive control, the TMMGP strain containing an empty vector was used as a negative control.

### Detection of Enantioselectivity

Racemic (*R*,*S*)-α-(4′-isobutylphenyl)-propionic acid methyl ester (*R*,*S*) and (*R*,*S*)-α-phenylpropionic acid ethyl ester (*R*,*S*) were prepared from the commercially available corresponding racemic acid by acid-catalyzed esterification with methanol and ethanol, respectively. The structure of the products was confirmed by ^1^H-NMR. For the biocatalysis reaction, racemic ester (11 mM) was dissolved in Tris-HCl buffer (50 mM, pH 7) with DMSO (10% v/v) as cosolvent. Purified PL-GOS (0.18 mg) was added to a total volume of 1 mL and the reaction mixture was incubated in a thermoshaker at 37°C and 900 rpm. The reaction was monitored by thin layer chromatography and was stopped after 120 h, at approximately 50% conversion. The reaction mixture was extracted twice with methyl tert butyl ether (MTBE, 0.4 mL). The organic solvent was dried over magnesium sulfate and removed under dry air. Substrate and product of the methyl ester hydrolysis were separated by TLC and the produced acid dissolved in MTBE. The acid products of both hydrolysis reactions were converted into the corresponding methyl esters with TMS-diazomethane (Sigma–Aldrich) according to the instructions of the manufacturer. The optical purity of the reactants was determined using a chiral gas chromatography column (Macherey-Nagel Hydrodex-ß-6TBDM, 25 m × 0,25 mm) in a Shimadzu GC-2010 gas chromatograph using hydrogen as carrier gas. Using 125°C column temperature, α-(4′-isobutylphenyl)-propionic acid methyl ester eluted at 14.50 min (*S*) and 14.80 min (*R*). Using 85°C column temperature, α-phenylpropionic acid methyl ester eluted at 15.80 min (*S*) and 16.30 min (*R*). The *E*-value of the biocatalysis of α-phenylpropionic acid ethyl ester were calculated using conversion according to gas chromatography and eeP. *E*-value and conversion of the biocatalysis of α-(4′-isobutylphenyl)-propionic acid methyl ester were calculated using eeS and eeP according to the equation from Chen et al. (1982).

## Results

### Bioinformatic Search for Lipolytic Enzymes

The GOS project is the biggest metagenomic project to date. It contains genomic data from planktonic microorganisms collected in oceanic surface water around the globe (Rusch et al., 2007). The 6,123,395 sequences of potential proteins from the GOS project were searched for potential novel lipolytic enzymes. In a first step, we bioinformatically assigned protein functions to these sequences using PfamA, PfamB, and TIGRFAM HMMs. Proteins that matched with an *E*-value below 10^-5^ were considered as “annotated”. These annotated sequences were removed from the protein sequence pool. The remaining 1.7 million sequences, which we considered of “unknown” function, were clustered at 90% similarity to avoid redundancy in the sequences. The resulting 706,208 sequences were then clustered using the markov-clustering algorithm MCL (van Dongen and Abreu-Goodger, 2011). This resulted in 17,795 clusters consisting of more than 10 sequences. We then identified one representative protein for each of these clusters. To do so, the protein with the minimal phylogenetic distance to all other proteins in the cluster was chosen. We argued that this protein best represents all other members of the cluster. We then pre-screened these representatives with a naïve motif-search for the consensus active site sequences of the three lipase superfamilies GX, GGGX, and Y (**Table 1**). This resulted in 470 sequences matching the search criteria for class GX, 149 sequences matching the search criteria for class GGGX and 158 sequences matching the search criteria for class Y. While the active site sequences are necessary but not sufficient for a lipase, we also used a HMM search for the hallmark alpha/beta hydrolase fold. This search was performed using HMMs describing the families of the Lipase Engineering Database (Pleiss et al., 2000). The protein gi135157357 (PL-GOS) from the GX-superfamily was the only sequence that matched with a significant *E*-value. The known lipase family closest to PL-GOS was abH15.03 (*Saccharomyces cerevisiae* lipase 2 like). A BLASTP comparison of PL-GOS and *Saccharomyces cerevisiae* lipase 2 showed an identity of 25, and 42% positive amino acid matches. The closest matches in the NCBI non-redundant sequence database using BLASTP were uncharacterized, hypothetical proteins of *Balneola vulgaris* and *Gracilimonas tropica* with an identity of 49% each and 69% and 68% positive amino acid matches, respectively. This potential lipase was then synthesized and cloned into our vector systems for periplasmic expression and overexpression.

### PL-GOS is a Lipase

**FIGURE 1:**
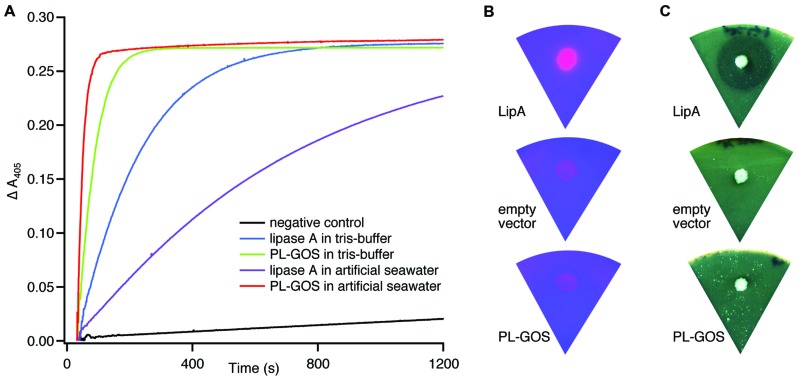
**Esterase activity of PL_GOS. (A)** Hydrolysis of pNP-butyrate by PL-GOS. Crude extract from *Escherichia coli* MG1655 cells expressing PL-GOS (strain TMMGP1503) and lipase A (strain TMMGPK) from *Bacillus subtilis* was used. Crude extract from *E. coli* TMMGP (empty vector) served as negative control. Activity of crude extracts was tested in tris-HCl-buffer, pH 7.3 and artificial seawater. PL-GOS activity increased in artificial seawater, while lipase A activity decreased. Plate based lipolytic assays with triolein/rhodamin **(B)** and tributyrate as substrate **(C)** show no lipase activity in *E. coli* TMMGP1503 expressing PL-GOS from a plasmid. *E. coli* TMMGPK expressing Lipase A from *B. subtilis* was used as a positive control and *E. coli* TMMGP with an empty vector was used as negative control.

To test this metagenomic protein for lipase or esterase activity, we tested activity in crude extract of *E. coli* MG1655 cells expressing PL-GOS from a plasmid. PL-GOS crude extract showed activity against pNP-butyrate similar to crude extract of MG1655 cells expressing *B. subtilis* lipase A, which we used as a positive control (**Figure 1A**). The activity of PL-GOS could be increased by exchanging the tris-HCl-buffer, pH 7.3 in the reaction solution for artificial seawater. This also resulted in lower activity of the positive control and suggested to us that PL-GOS could have halotolerant properties.

From the general potential of PL-GOS to hydrolyze organic ester bonds in substrates with long fatty acid chains like pNP-palmitate, we concluded that PL-GOS is a lipase, though direct hydrolytic activity toward triglycerides could not be shown in our assays (**Figures 1B,C**).

### PL-GOS has a Broad Range of Substrate Chain Length

**FIGURE 2:**
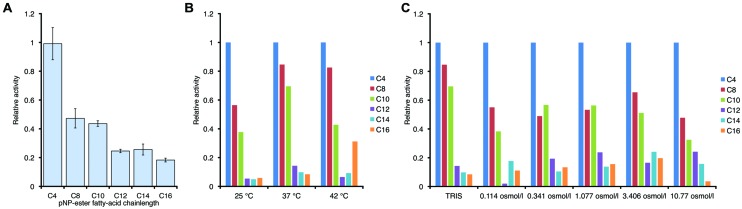
**Chain length specificity of the hydrolase activity of PL-GOS. (A)** pNP esters with different acid residue chain length where tested at 20°C in tris-HCl-Buffer, pH 7.3. Enzyme concentration was at 65 nM, substrate concentration was 200 μM. Activity is expressed relative to activity toward pNP-butyrate. Relative preference for shorter chain length did not change with temperature, as tested in tris-HCl-Buffer **(B)** or osmolarity of the buffer, as tested at 37°C **(C)**. Experiments for **(B)** and **(C)** were conducted once.

We therefore decided to determine the biochemical characteristics of this biocatalyst in more detail. By attaching an N-terminal His_6_-tag we were able to purify PL-GOS to 95% homogeneity. Esterases typically prefer hydrophilic substrates and thus generally show a lower activity toward substrates with longer carbon chains. Some esterases do not show any activity toward pNP esters with a chain length longer than C_8_ (Yu et al., 2010), while others have a maximum activity toward pNP-C_8_ and lower activity toward shorter and longer chained pNP-esters (Choi et al., 2004). The chain length specificity of PL-GOS for the hydrolysis of pNP-esters was determined by using pNP-esters with variable acid residue lengths ranging from butyrate (C4) to palmitate (C16). PL-GOS exhibited its highest activity toward pNP-butyrate. It showed a decrease of activity with increasing length of the acid residue chain. The activity toward pNP-octanoate (C8) was 47.6%, the activity toward pNP-palmitate (C16) 13.3% of the activity toward the preferred pNP-butyrate (C4) substrate (**Figure 2**). This indicates that while PL-GOS prefers shorter chains, it hydrolyzes a broad spectrum of substrate chain lengths, which is typical for some lipases.

### Enzyme Kinetics of PL-GOS

**FIGURE 3:**
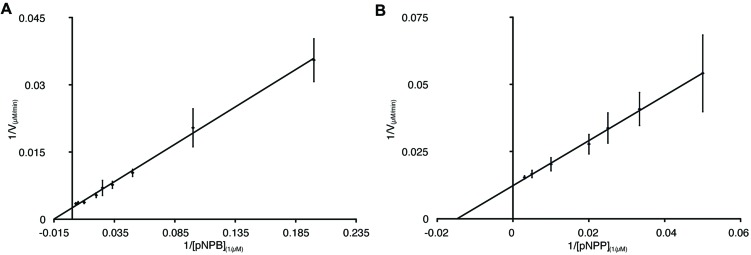
**Enzymatic parameters of PL-GOS.** K_m_ and V_max_ toward pNP-butyrate **(A)** and pNP-palmitate **(B)** were derived from Lineweaver–Burk plots with a linear fit. The x-axis displays 1/[substrate] in μM^-1^. For pNP-butyrate the K_m_ was 66.68 μM and V_max_ was 400 μM/min. The apparent K_m_ of pNP-palmitate was 24.18 μM with a V_max_ of 50.76 μM/min. Enzyme concentration was 65 nM, assays were performed at 20°C.

To further characterize the activity of PL-GOS, its Michaelis Menten kinetics toward pNP-butyrate and pNP-palmitate were determined (**Table 3**). The K_M_ of PL-GOS toward pNP-butyrate was 66.68 μM. From our experiments we derived a *k*_cat_ value of 228 s^-1^ and a *k*_cat_/*K*_M_ value of 3.4 * 10^6^ M^-1^s^-1^ (**Figure 3A**). Compared to esterases which are specialized to hydrolize ester-bonds with short-chain fatty acids, PL-GOS shows a very high affinity for its preferred substrate, but a comparatively low V_max_ (Choi et al., 2004; Lee et al., 2006; Yu et al., 2010). Toward pNP-palmitate, the apparent K_M_ of PL-GOS was measured to be similar with 68.08 μM at substrate concentrations above 20 μM. At lower concentrations of pNP-palmitate PL-GOS displayed non-Michaelis Menten kinetics and the apparent K_M_ was lower. This might be due to the propensity of the substrate to form micelles (see Discussion, **Figures 6** and **7**). The *k*_cat_ value at substrate concentrations above 20 μM, on the other hand was significantly lower at 45 s^-1^, when compared to pNP-butyrate, resulting in a *k*_cat_/*K*_M_ value of 6.6 * 10^5^ M^-1^s^-1^ (**Figure 3B**). The high activity toward long-chain fatty acids suggests that PL-GOS is a lipase. Some other lipases show a significantly lower affinity toward pNP-palmitate when compared to PL-GOS. The lipase of *Burkholderia cepia*, for example, has a K_M_ toward pNP-palmitate of 12 mM (Sharma et al., 2001).

**Table 3:** Enzymatic properties of PL-GOS.

Property	pNP-butyrate	pNP-palmitate
(apparent) K_m_ (μM)	66.68	68.08
V_max_ (μM/min)	400	50.76
k_cat_ (s^-1^)	228	45
k_cat_/K_m_ (M^-1^s^-1^)	3.4 * 10^6^	6.6 * 10^5^
	
Temperature optimum	19.85°C	
Osmolarity optimum	3.89 osmol/l	

### PL-GOS Shows Moderate Enantioselectivity

**FIGURE 4:**
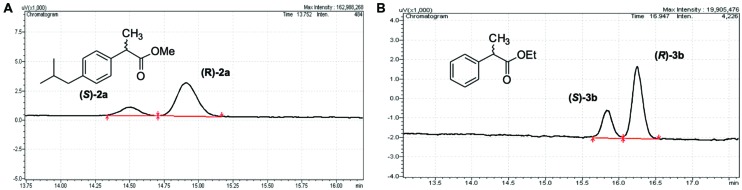
**Enantioselectivity of PL-GOS.** PL-GOS preferably hydrolyzes the R-enantiomers of Ibuprofen methyl ester **(A)** and phenylpropionic acid methyl ester **(B)**. Racemic mixtures of the respective esters were incubated with PL-GOS, then products were derivatized with TMS-diazomethane and separated on a chiral gas chromatography column.

The lipase-catalyzed kinetic resolution of racemic chiral esters is a widely used route for the synthesis of optically pure compounds. To investigate a potential application of PL-GOS, we studied the kinetic resolution of two racemic esters. Ibuprofen is a non-steroidal inflammatory drug and is one of the most-sold over-the-counter drugs (Kourist et al., 2011). Biocatalysis offers an environmentally friendly approach for the preparation of these compounds in optically pure form. Phenylpropionic acid is a chiral building block for pharmaceutical synthesis and is a model compound for the conversion of profens. PL-GOS showed promising enantioselectivity in the kinetic resolution of esters of these compounds. It shows preference toward the (*R*)-enantiomers and enantioselectivity of *E* = 3–4, which allows synthesis of the products in enantioenriched form (43%ee for Ibuprofen methyl ester and 61%ee for the ethyl ester of phenylpropionic acid; **Figure 4**). This moderate selectivity suggests a potential of PL-GOS as enantioselective biocatalyst.

### PL-GOS is a Phsychrophilic and Halotolerant Enzyme

**FIGURE 5:**
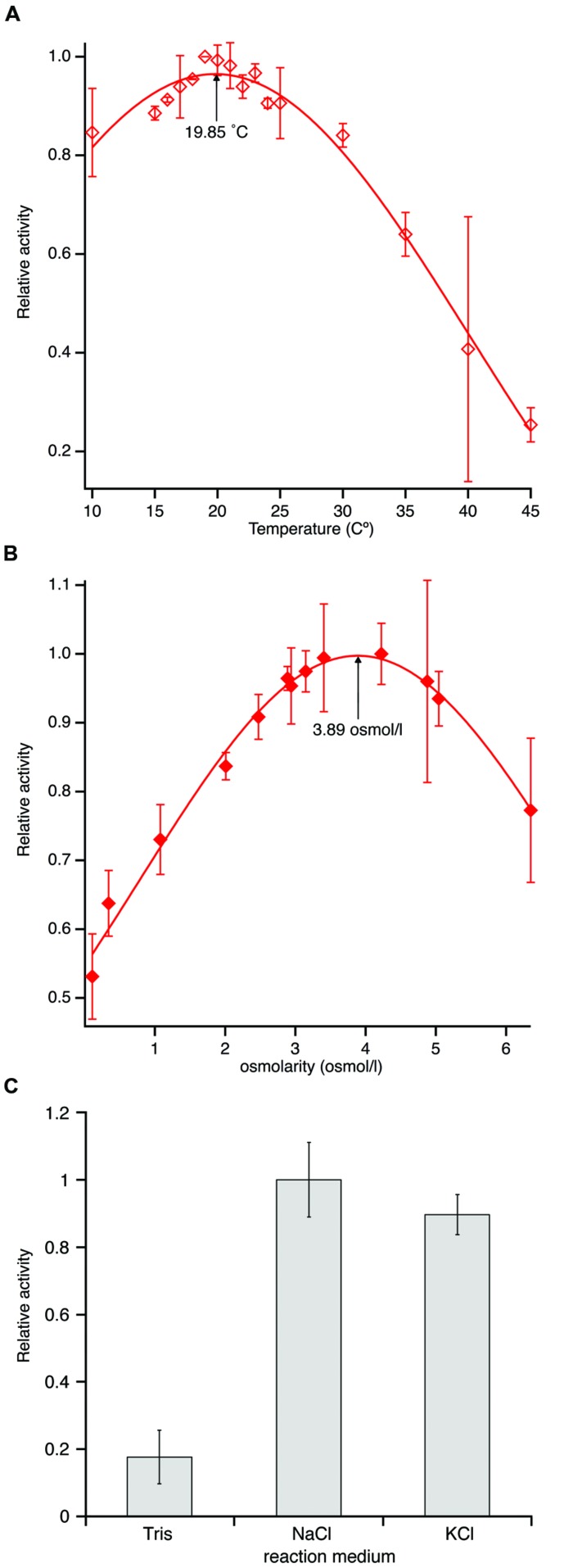
**Temperature and osmolarity optimum of PL-GOS using pNP-butyrate as substrate. (A)** Activity was measured at different temperatures between 10 and 45°C in tris-HCl-buffer, pH 7.3 and set in relation to the highest activity. An optimum temperature of 19.85°C was calculated by gaussian curve fitting. **(B)** Activity of PL-GOS at 19.85°C in solutions with different osmolarities made by adjusting NaCl concentrations in artificial seawater and set in relation to the highest activity. An optimum of 3.89 osmol/l was calculated using gaussian curve fitting. **(C)** Influence of alkali metal cations on the activity of PL-GOS: Artificial seawater with three times higher osmolarity than standard was prepared by the addition of either NaCl or KCl. No significant difference between the cationic composition of the buffer was found. Activity in Tris-HCl for comparison.

Due to its maritime origin we speculated that PL-GOS would be most active at mild to cold temperatures. The activity of PL-GOS was therefore measured at different temperatures in the range between 10°C and 45°C. From this data the temperature optimum could be interpolated to be 19.85°C (**Figure 5A**; **Table 3**), while still retaining 80% of its activity at 10°C. At higher temperatures its activity diminished and at 45°C it showed only a quarter of its maximum activity.

Another characteristic, which we speculated PL-GOS could possess due to its maritime origins, was a tolerance or affinity for high salt concentrations. In our crude extract assays we already determined that PL-GOS has a higher activity in artificial seawater when compared to tris-HCl-buffer, pH 7.3. The osmolarity optimum was therefore determined next. To do this, different reaction buffers with varying NaCl concentrations, derived from artificial seawater, were used in a pNP-butyrate hydrolysis assay. This revealed an osmolarity optimum of 3.89 osmol L^-1^, which amounts to 3.61-fold the osmolarity of artificial seawater (**Figure 5B**; **Table 3**). Dependency of the increased activity on Na^+^-ions was tested by interchanging NaCl with KCl. No significant difference between reaction medium with a high concentration of NaCl or a high concentration of KCl could be detected (**Figure 5C**).

## Discussion

We present here a combined bioinformatics/biochemistry approach for the discovery of new biocatalytic enzymes from metagenomic datasets. The amount of proteins encoded in the DNA of microbial communities is enormous. In a typical metagenomic discovery project it is therefore necessary to create large expression libraries with 100s of 1000s of clones and screen this library for a specific enzyme activity. These projects have generated many new biocatalysts (Coughlan et al., 2015). However, this process is resource- and time-consuming and often yields only a low hit per clone ratio typically in the range of 10^-6^ to 10^-4^ depending on the size of the DNA fragments used (Lämmle et al., 2007). This low ratio is not necessarily the result of a low frequency of the occurrence of proteins with the screened-for activity, but also based on the imperfect positioning of the genes in the expression library. With the advent of high-throughput “next-generation” sequencing, a bioinformatics approach can substitute the creation and screening of an expression library. This approach allows for a first “screening” *in silico*. Once established, the bioinformatical screening process can be applied to further metagenomic datasets with little to no additional cost. However, high-throughput bioinformatics cannot yet annotate every potential protein correctly (Godzik, 2011). Thus, one disadvantage of a discovery strategy solely based on a bioinformatic homology search is the dismissal of protein families with a low homology to known biocatalysts. Here we present an approach to find a biocatalyst through systematic application of different bioinformatics search methods to a metagenomic dataset.

**FIGURE 6:**
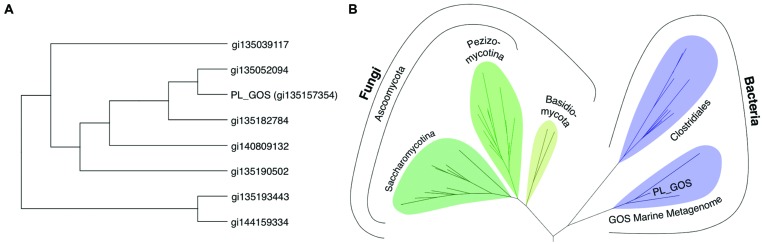
**Phylogenetic analysis of the metagenomic sequences of the cluster of potential lipases containing PL-GOS. (A)** Unrooted phylogenetic tree of all eight complete, non-redundant sequences of the PL-GOS cluster. **(B)** Phylogenetic tree of lipase sequences from family abH 15.03 from the Lipase Engineering Database and the PL-GOS cluster. The PL-GOS cluster forms a discrete subfamily in the bacterial branch of abH 15.03. Sequences from family abH 15.02 were used as an outgroup to define the root.

**FIGURE 7:**
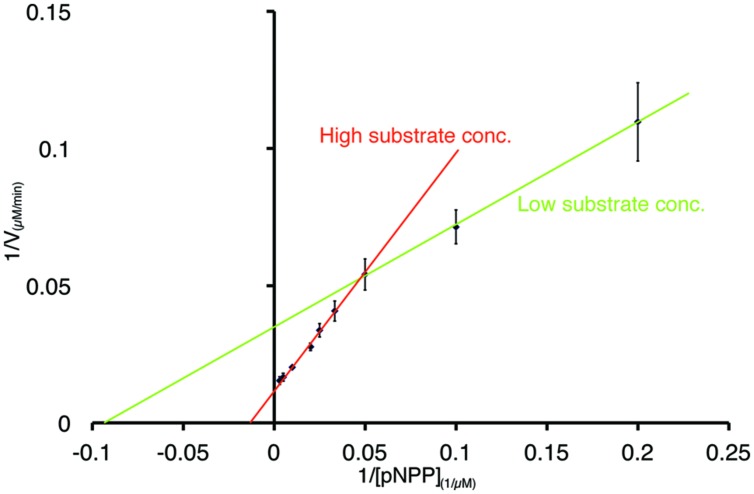
**Non-Michaels Menten kinetics of PL-GOS at low substrate concentrations of pNP-palmitate.** Lineweaver–Burk plot of PL-GOS enzyme kinetics with pNP-palmitate as substrate. At low concentrations (≤20 μM), the apparent K_m_ of PL-GOS was 10.64 μM, at higher concentrations the K_m_ was higher at 68.08 μM.

We identify the novel lipase PL-GOS, representative member of a cluster of 27 potential lipase sequences in the GOS dataset. The genes with an unambiguously predicted start and stop codon in this cluster encode eight complete proteins (**Figure 6A**), the rest encodes partial protein sequences. A phylogenetic analysis revealed that this cluster forms a discrete bacterial subfamily of the abH15.03 (*Saccharomyces cerevisiae* lipase 2 like) family from the Lipase Engineering Database (**Figure 6B**). PL-GOS a phsychrophilic and halophilic lipase. Especially toward substrates with long carboxylic acid chains PL-GOS showed a favorable activity when compared to other carboxylester hydrolases (Sharma et al., 2001; Choi et al., 2004; Lee et al., 2006; Yu et al., 2010). Due to the propensity of pNP-palmitate to form micelles, the determination of kinetic parameters for this substrate is challenging. Indeed, the apparent K_M_ at very low concentrations of pNP-palmitate was measurably lower than with high and medium concentrations (**Figure 7**).

**FIGURE 8:**
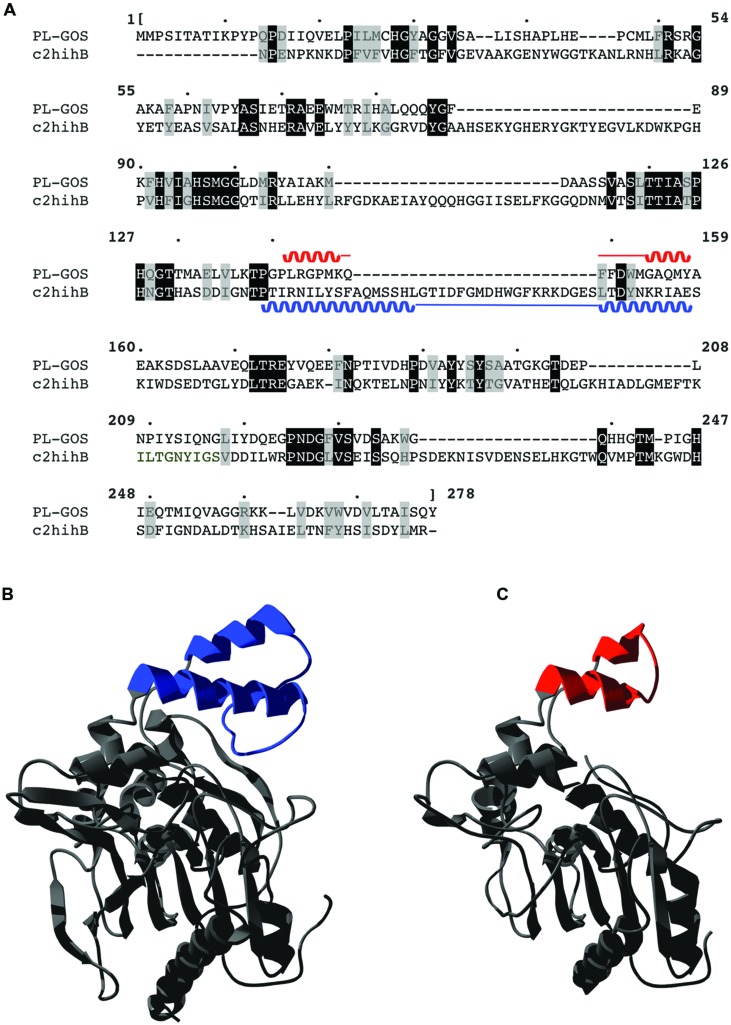
**Predicted lid structure of PL-GOS.** PL-GOS was aligned with the lipase of *Staphylococcus hyicus* (c2hihB in the PDP database), a lipase with a known 3D-structure and 22% identity with PL-GOS **(A)**. The known structure of *S. hyicus* lipase **(B)** was used for 3D modeling of PL-GOS by Phyre2 **(C)**. The lid structures are highlighted in blue and red, respectively. In case of PL-GOS the predicted lid structure is truncated.

A Phyre2 alignment to predict the 3D structure of PL-GOS showed highest similarity to a *Staphylococcus hyicus* lipase, which is descriped to have a lid structure (Tiesinga et al., 2007). The predicted 3D-structure of PL-GOS shows a truncated lid (**Figure 8**) which further points to PL-GOS being a lipase. However, although the Phyre2 confidence score for the structural model is high (at 100.0), its prediction is based on weak homology (22% sequence identity), so its validity can be questioned. Furthermore, no activity toward triglycerides could be detected in our plate assays.

The commercial viability of biocatalysts depends on many factors and advances in protein engineering in recent years have shown great success in enhancing enzyme characteristics and specializing their uses for industrial application in biocatalysts as a whole (Bornscheuer et al., 2012; Li and Cirino, 2014) and carboxylesterases in particular (Widersten, 2014). The very mild temperature requirements and interesting osmolarity dependencies of PL-GOS in combination with its broad substrate range could form the basis of protein engineering enterprises.

## Conflict of Interest Statement

The authors declare that the research was conducted in the absence of any commercial or financial relationships that could be construed as a potential conflict of interest.
